# Choice as a Meaning-Making Device for Maximizers: Evidence From Reactance to Restrictions of Choice Freedom During Lockdown

**DOI:** 10.3389/fpsyg.2020.571462

**Published:** 2020-11-09

**Authors:** Michail D. Kokkoris

**Affiliations:** Vrije Universiteit Amsterdam, Department of Marketing, Amsterdam, Netherlands

**Keywords:** maximizing, search for meaning, existentialism, identity, reactance, online shoping, lockdown, COVID-19 pandemic

## Abstract

The current research investigates maximizers’ responses to restrictions of choice freedom during lockdown in the context of the COVID-19 pandemic. Having as a starting point the assumption that for maximizers choice is constitutive of identity, this research proposes that maximizing is associated with search for existential meaning in life. In turn, maximizers’ propensity to search for meaning is associated with a higher susceptibility to experience reactance when their freedom of choice is restricted, which is further associated with higher engagement in online shopping during lockdown presumably as a means to combat reactance and restore choice freedom. Using the lockdown in spring 2020 as a naturalistic context to study consumer responses to restrictions of choice freedom, results of an online study in Austria support these predictions. These findings advance a view of maximizers as “lay existentialists,” who view choice as a meaning-making device that is tightly linked to their sense of identity. As a result, when their choice freedom is threatened, maximizers may respond with higher reactance and engage in restorative actions.

## Introduction

In the past two decades, research on individual differences in decision-making has shed much light on how maximizing – the tendency to strive for the best choice – relates to various spheres of life (for reviews, see Cheek and Schwartz, 2016; Misuraca and Fasolo, 2018). Despite considerable advances, not much is known about the way maximizers respond to restrictions of their choice freedom and what role choice plays more generally in maximizers’ lives. The COVID-19 pandemic provides a naturalistic context to study maximizers’ consumer behavior under limited choice due to the lockdowns implemented in spring 2020 in many countries around the world.

This research proposes that maximizers are individuals in pursuit of existential meaning. Existential meaning can be defined as “the sense made of, and significance felt regarding, the nature of one’s being and existence” (Steger et al., 2006, p. 81). Drawing on recent research suggesting that maximizers are oriented toward the future (Misuraca et al., 2016; Zhu et al., 2017), achievement (Peng et al., 2018), and self-fulfillment (Kokkoris, 2016), the current research proposes that the quest for the best choice is associated also with a broader quest for the meaning of existence. Why would searching for the best choice be associated with searching for meaning in life? A potential answer is because choice for maximizers is an act of meaning that is constitutive of identity. For maximizers, every choice they are making, from the smallest to the biggest, defines who they are, shapes their existence, and ultimately can give their lives meaning.

A vast amount of research in social psychology has long shown that choice, besides being “contemplation of alternatives and selection among them” (Vohs et al., 2008, p. 884), is also an act that reifies the self by expressing inner aspects of the self, such as preferences, attitudes, values, and beliefs (Iyengar and Lepper, 1999; Tafarodi et al., 2002; Kim and Drolet, 2003; Snibbe and Markus, 2005; Kokkoris and Kühnen, 2015). Research in sociology has also shown that choice plays an even more crucial role for identity formation in modernity, because nowadays identities are not fixed or inherited but rather shaped through choosing for oneself who one wants to be (Giddens, 1991; Inglehart and Oyserman, 2004; Salecl, 2010). In addition, according to the philosophical tradition of existentialism, choice gives meaning to one’s life. For example, Jean-Paul Sartre argued that it is through choice that one’s existence becomes meaningful (Grisoli, 1945/2009, p. 16). Relatedly, Søren Kierkegaard declared that choice is “the act by which an individual may become a person” (Stack, 1973, p. 112). According to this school of thought, our lives do not just contain our choices; our lives are our choices. Maximizers are individuals who are particularly suited to subscribe to this view. As they strive to make the best choice across domains, from the most mundane to the most consequential (Kokkoris, 2019), they might also be prone to consider choice as constitutive of identity. Through individual choice, they search for answers to the big existential questions in life.

If individuals high (vs. low) in maximizing search more for meaning in life driven by a view of choice as paramount to identity construction and expression, then they might also experience more reactance when a situation does not afford them unconstrained choice. A classical finding in social psychology is that when people feel that any of their free behaviors is eliminated or threatened with elimination, they experience an unpleasant motivational arousal, which is called reactance (Miron and Brehm, 2006). Although reactance is a common response of all people to restrictions of freedom, maximizers are expected to be particularly sensitive to such restrictions because, as reactance theory postulates, the intensity of reactance depends on the importance of the threatened freedom (Miron and Brehm, 2006). The importance of choice freedom can be considered to be higher for maximizers, because choice for them does not serve only functional needs but also existential purposes. Thus, limitations of choice freedom might induce higher reactance among individuals high rather than low in maximizing. The COVID-19 pandemic provided a fitting setting to test this. In spring 2020, many countries around the world implemented lockdowns in order to contain the spread of the new coronavirus. With most shops closed, freedom of movement strictly regulated, and oftentimes stockpiling leading to shortage of goods even in shops that remained open like supermarkets and pharmacies, consumer choice during lockdown has been arguably drastically limited. This allows for the study of maximizers’ response to restrictions of choice freedom in a naturalistic context.

Finally, this research also examines an outcome related to consumer behavior. Theory posits that the aversive motivational state of reactance can result in behaviors that attempt to reestablish the freedom that has been eliminated (Miron and Brehm, 2006). In this case, one way to reestablish choice freedom could be by engaging more in online shopping, which continued to be available during lockdown. Ordering consumer products online could be a way to bypass limitations posed on choice freedom and restore feelings of unconstrained choice. Thus, if maximizers experienced more reactance, they might have engaged in online shopping during lockdown more than they would normally do in other times.

In short, the current research examines whether individuals high (vs. low) in maximizing are more likely to (a) view choice as identity, (b) search for meaning in life, (c) experience consumer reactance when choice freedom is limited, and eventually (d) engage more in online shopping during lockdown as a way to restore freedom of choice. One pilot study and one main study test the above predictions.

## Pilot Study

A pilot study first tested the underlying assumption of this research that for individuals high (vs. low) in maximizing choice is more tightly tied to identity.

### Materials and Methods

#### Participants

The association between maximizing and choice as identity was pilot-tested in two samples: a United States community sample (*N* = 132) recruited from prolific for monetary compensation (81 men, 51 women, age 18–74, *M* = 32.64, *SD* = 13.05) and a European student sample (*N* = 167) recruited from a subject pool of a large Austrian university for course credit (80 men, 87 women, age 18–30, *M* = 21.59, *SD* = 2.28). A sensitivity power analysis showed that the respective sample sizes can reliably detect small to medium effect sizes of *ρ* = 0.21 and *ρ* = 0.19 (one-tailed) with an alpha level of 0.05 and power of 0.80.

#### Procedure

Both studies were conducted before the COVID-19 pandemic. Participants in the United States took the study online, whereas participants in Austria took the study in the lab. Participants first filled out the Maximizing Tendency Scale (Diab et al., 2008). It consists of nine items (e.g., “No matter what it takes, I always try to choose the best thing”) on a 7-point scale (1 = *strongly disagree* and 7 = *strongly agree*). This scale has been recommended as the most suitable measurement of the maximizing construct among the various available alternatives (Cheek and Schwartz, 2016), because it does not confound maximizing with decision difficulty (Diab et al., 2008), as the original Maximization Scale for instance does (Schwartz et al., 2002). In both samples, maximizing had very good reliability (*α* = 0.87 in the United States sample and *α* = 0.80 in the Austrian sample). Then, participants filled out a measure of choice as identity that was devised for the purpose of this research (see Table 1 for details). It consists of six items (e.g., “My choices are an important part of my identity”) on a 7-point scale (1 = *strongly disagree* and 7 = *strongly agree*). In both samples, all items loaded on a single factor (explaining 66.17% and 64.48% of the total variance in the United States and the Austrian sample, respectively). Hence, a composite score of choice as identity was created (*α* = 0.89 in both samples).

**Table 1:** Items and factor loadings for choice as identity.

	Item	Factor loadings		
		Sample 1	Sample 2	Sample 3
1.	What people choose shows who they are.	0.84	0.80	0.70
2.	Every choice, no matter how small or trivial, is an act of self-expression.	0.80	0.80	0.79
3.	Choice makes a statement about the kind of person one is.	0.83	0.86	0.75
4.	Compared to other means of self-expression (e.g., our thoughts, feelings, ideas, and beliefs), our choices say the most about ourselves.	0.74	0.72	0.86
5.	My choices are an important part of my identity.	0.87	0.86	0.81
6.	We are the sum of our choices.	0.79	0.76	0.83

Sample 1: Pilot study (United States); Sample 2: Pilot study (Austria); Sample 3: Main study (Austria). The factor analysis was a principal-components analysis with varimax rotation and eigenvalues greater than 1 as the extraction method. Instructions given to the participants together with these items were: *“Choice is whenever people evaluate alternatives and make a selection among two or more options. Here, we refer to all kinds of choices, from the smallest (clothes, foods, entertainment, etc.) to the biggest (studies, jobs, partners, etc.). Please indicate how much you agree with each statement.”* Items were rated on a 7-point scale (1 = *strongly disagree*; 7 = *strongly agree*).

### Results

Results showed a significant positive correlation between maximizing and choice as identity both in the United States sample, *r* = 0.36, 95% CI = (0.184, 0.536), *p* < 0.001, and in the Austrian sample, *r* = 0.34, 95% CI = (0.158, 0.517), *p* < 0.001. Using data from two different samples, the pilot study provides convergent evidence that individuals high (vs. low) in maximizing are more likely to construe choice as constitutive of identity.

## Main Study

### Materials and Methods

#### Participants

One-hundred and thirty-nine undergraduate students of a large Austrian university were recruited *via* the university subject pool and took part in the study online for course credit. Two participants failed an attention check (to select a specific answer in one question) and were excluded from further analyses. The final sample comprised 137 participants (49 men, 88 women, age 20–37, *M* = 22.36, *SD* = 2.32). A sensitivity power analysis showed that this sample size can reliably detect small to medium effect sizes of *ρ* = 0.21 (one-tailed) with an alpha level of 0.05 and power of 0.80.

#### Procedure

The study was conducted after the lockdown in Austria was lifted (specifically on May 12–15, 2020). Participants first completed the same measure of maximizing (*α* = 0.81) as in the pilot study. Then, they filled out the Meaning in Life Questionnaire (Steger et al., 2006), which consists of two subscales with five items each (1 = *absolutely untrue* and 7 = *absolutely true*): presence of meaning (*α* = 0.89; e.g., “I have a good sense of what makes my life meaningful”) and search for meaning (*α* = 0.90; e.g., “I am looking for something that makes my life feel meaningful”). Choice as identity (*α* = 0.88) was assessed with the same six items as in the pilot study (all items loaded again on a single factor explaining 62.67% of the total variance). Consumer reactance during lockdown (*α* = 0.91) was assessed with the following five items on a 7-point scale (1 = *strongly disagree* and 7 = *strongly agree*) based on the reactance literature (Hong, 1992; Jonas et al., 2009): “During the recent lockdown due to the coronavirus … I often felt that I had limited choices as a consumer,” “…I often felt very restricted as a consumer,” “…I was often frustrated that I was unable to make free consumer choices,” “…I was often distressed that I could not have what I wanted as a consumer,” and “…I was often irritated that many consumer options were no longer available.” Finally, online shopping during lockdown was assessed with a question asking participants to indicate whether during lockdown they: (a) started doing online shopping for the first time, (b) did online shopping more than before, (c) did online shopping as much as before, (d) did online shopping less than before, or (e) did not do any online shopping at all.

### Results

First of all, inspection of correlation coefficients confirms the main predictions of this study. Specifically, maximizing was associated with (a) search for meaning, *r* = 0.27, 95% CI = (0.080, 0.451), *p* = 0.002; (b) viewing choice as identity, *r* = 0.26, 95% CI = (0.087, 0.410), *p* = 0.002; and (c) experiencing consumer reactance, *r* = 0.17, 95% CI = (0.008, 0.316), *p* = 0.051 (descriptive statistics and inter-correlations of all variables are presented in Table 2).^1^ Regarding online shopping, 2 participants (1.5%) reported starting online shopping for the first time during lockdown, 35 participants (25.5%) reported doing more online shopping than before, 66 participants (48.2%) doing as much online shopping as before, 14 participants (10.2%) doing less online shopping than before, and 20 participants (14.6%) not doing online shopping at all. Individuals who reported doing more online shopping during lockdown than before (*M* = 5.17, *SD* = 0.74) tended to score higher on maximizing than all other participants (*M* = 4.89, *SD* = 0.89), *t*(135) = 1.70, *p* = 0.091.

**Table 2:** Descriptive statistics and inter-correlations.

	1	2	3	4	5
1. Maximizing					
2. Presence of meaning	0.10 (−0.06, 0.26)				
3. Search for meaning	0.27^***^ (0.08, 0.45)	−0.20^**^ (−0.38, −0.01)			
4. Choice as identity	0.26^***^ (0.09, 0.41)	0.06 (−0.10, 0.21)	0.23^***^ (0.08, 0.37)		
5. Consumer reactance	0.17^*^ (0.01, 0.32)	−0.10 (−0.26, 0.06)	0.26^***^ (0.12, 0.40)	0.06 (−0.10, 0.22)	
Cronbach’s alpha	0.81	0.89	0.90	0.88	0.91
*M*	4.96	4.79	5.00	5.07	3.56
*SD*	0.86	1.18	1.25	0.98	1.40
Min	2.33	1.80	1.00	2.00	1.00
Max	6.67	7.00	7.00	7.00	6.60

BCa bootstrap 95% CIs (1,000 samples) reported in brackets.

*** *p* < 0.01;

** *p* < 0.05;

* *p* < 0.10.

**Figure 1:**
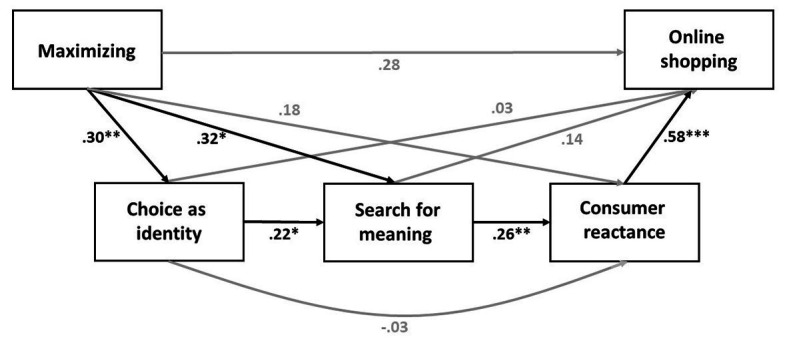
Conceptual model results. ^***^p < 0.001; ^**^p < 0.01; and ^*^p < 0.05. Unstandardized coefficients are provided along the paths. The indirect effect of maximizing on online shopping *via* choice as identity, search for meaning, and consumer reactance: *B* = 0.0100, *SE (Boot)* = 0.0083, 95% CI = (0.0004, 0.0321). All other models with alternative orders of the variables were not significant: search for meaning – reactance – choice as identity: *B* = 0.0000, *SE (Boot)* = 0.0017, 95% CI = (−0.0032, 0.0038); reactance – choice as identity – search for meaning: *B* = 0.0001, *SE (Boot)* = 0.0011, 95% CI = (−0.0018, 0.0029); reactance – search for meaning – choice as identity: *B* = 0.0002, *SE (Boot)* = 0.0027, 95% CI = (−0.0040, 0.0069); search for meaning – choice as identity – reactance: *B* = −0.0009, *SE (Boot)* = 0.0049, 95% CI = (−0.0122, 0.0084); and choice as identity – reactance – search for meaning: *B* = 0.0002, *SE (Boot)* = 0.0023, 95% CI = (−0.0034, 0.0059).

Next, the entire path model (maximizing → choice as identity → search for meaning → consumer reactance → online shopping) was tested (PROCESS model 6; Hayes, 2013). Maximizing was entered as independent variable, choice as identity as serial mediator 1, search for meaning as serial mediator 2, consumer reactance as serial mediator 3, and online shopping as dependent variable (dummy coded; 1 = more than before and 0 = all other answers). Model fit results show that the four-variable model fits better than the constant-only model, *χ*^2^(4) = 19.03, *p* < 0.001, McFadden pseudo-*R*^2^ = 0.122. Specifically, results showed that maximizing was associated with viewing choice as identity, *B* = 0.30, *SE* = 0.09, *p* = 0.002, 95% CI = (0.112, 0.487). In turn, choice as identity was associated with search for meaning, *B* = 0.22, *SE* = 0.11, *p* = 0.044, 95% CI = (0.006, 0.433). Search for meaning was associated with consumer reactance, *B* = 0.26, *SE* = 0.10, *p* = 0.008, 95% CI = (0.069, 0.459). Finally, consumer reactance was associated with more online shopping during lockdown, *B* = 0.58, *SE* = 0.17, *p* = 0.001, 95% CI = (0.254, 0.902). The direct effect of maximizing on online shopping after controlling for choice as identity, search for meaning, and consumer reactance was not significant, *B* = 0.28, *SE* = 0.29, *p* = 0.340, 95% CI = (−0.292, 0.846). Critically, the indirect effect of maximizing on online shopping *via* choice as identity, search for meaning, and consumer reactance was significant, *B* = 0.010, *SE (Boot)* = 0.008, 95% CI = (0.0004, 0.0321; see Figure 1 for the entire model). Moreover, none of the alternative models with the mediator variables in different positions produced significant results (see note in Figure 1).

## Discussion

The current research suggests that maximizers are individuals that search for meaning in life and do so by investing their identities in the choices they make. In turn, maximizers’ pursuit of existential meaning is associated with reactance when limitations are imposed on their freedom of choice. Using the COVID-19 pandemic as a naturalistic context to study responses to restrictions of consumer choice, the results provide support to the hypotheses. Moreover, maximizers’ higher reactance to limitations of choice freedom predicted more engagement in online shopping during lockdown, presumably as a way to combat reactance and restore choice freedom.

These findings contribute to the decision-making literature on individual differences in maximizing by showing that maximizers are searching for meaning in life through the choices they make. Against this backdrop, maximizers can be conceptualized as “lay existentialists.” They are individuals who live by the moto “we are the sum of our choices.” For maximizers, choice is not just a functional tool to get what they want; it is also a meaning-making device with a profound existential impact. In that respect, limitations of choice freedom might be akin to an existential threat for maximizers. They experience reactance because limitations to their freedom of choice impede their existential pursuits to answer the big questions about life, identity, and existence. Consequently, they are ready to take action when their freedom of choice is threatened.

An ongoing debate in the maximizing literature is whether maximizing is beneficial or detrimental for well-being (e.g., Kokkoris, 2016; Vargová et al., 2020). What can the current finding about the association of maximizing with search for meaning tell us about this? As in prior research (Steger et al., 2006), presence of meaning and search for meaning correlated negatively with each other. Search for meaning can be both beneficial (Steger et al., 2008; Boyraz et al., 2010) and detrimental (Linley and Joseph, 2011) for well-being, whereas presence of meaning is more unambiguously considered as beneficial (for a review, see Linley and Joseph, 2011). Maximizing correlated positively with search for meaning and was uncorrelated (in fact correlated positively but not significantly) with presence of meaning. Therefore, one cannot say whether these findings clearly speak for the bright or the dark side of maximizing. What can be told for sure is that maximizers do not search for meaning because they suffer from an existential void. If that was the case, maximizing should have been positively associated with search for meaning and negatively associated with presence of meaning. The fact that maximizing is positively associated with search for meaning and uncorrelated with presence of meaning implies that maximizers’ tendency to search for meaning – regardless of whether they have already found meaning in life – is an indication of a genuinely inquisitive personality rather than a lack of meaning and despair.

Although not a primary focus of this research, an interesting side finding is that search for meaning was positively associated with consumer reactance. One could speculate that this is because choice freedom is a prerequisite for any kind of unobstructed search (not only for meaning). In that respect, people who search for meaning in life might experience stronger consumer reactance because any restrictions to their choice freedom, even in the consumption domain, are perceived as barriers to their search endeavors. An interesting avenue for future research would be to examine whether search for meaning is associated with a higher valuation of choice freedom in general and how this is manifested in various choice domains beyond consumption (interpersonal, professional, educational, etc.). Moreover, it might seem paradoxical at first glance that people search for meaning in consumption, given that materialism is known to be associated with lower well-being and meaning in life (Kashdan and Breen, 2007). But the distinction between presence of meaning and search for meaning is crucial here. Whereas people who have already found meaning in life might rely less on consumerism, people who still search for meaning might have hopes that consumerism can give their lives meaning. Indeed, in this study, consumer reactance was negatively (although not significantly) associated with presence of meaning but positively associated with search for meaning. Even though materialism apparently does not give life meaning, people who search for meaning probably consider the consumption domain as a potential source of meaning. This could be one more case of affective misforecasting like many others documented in the consumer research literature (e.g., MacInnis et al., 2005). Future research could explore this point further.

It is important to note that these conclusions are based on correlational data in a very specific context of the COVID-19 pandemic. As much as the lockdown might have served as a fitting naturalistic laboratory to study the current research questions, further research in other contexts, times, and populations is necessary in order to draw safer conclusions about the relationship between maximizing, meaning, and reactance. The pilot study, which tested the association of maximizing with choice as identity in two different populations before the pandemic, partly provides some evidence for the robustness of the results. However, the association with search for meaning and reactance needs to be further validated beyond the current historical context. It should also be noted that, although the sample size provided sufficient power to test the predictions, all effects in this research were of small to medium size. Furthermore, given that the use of different scales has been shown to produce strikingly different patterns of results (e.g., Cheek and Schwartz, 2016; Misuraca and Fasolo, 2018), future research could examine whether these conclusions hold with different conceptualizations and measurements of the maximizing construct. Finally, whereas the cross-sectional nature of the data clearly does not allow for any causal claims, the reported mediation analyses tested theoretically meaningful links between the variables. Although it is reasonable to treat maximizing as the predictor variable and other, more transient constructs (such as consumer reactance or online shopping during lockdown) as potential outcomes, other relations between the variables, not tested here, are also plausible. Future research using experimental or longitudinal designs is needed to test the causal relations between these variables.

## Data Availability Statement

The raw data supporting the conclusions of this article are available at: https://osf.io/mx35z/.

## Ethics Statement

Ethical review and approval was not required for the study on human participants in accordance with the local legislation and institutional requirements. The patients/participants provided their written informed consent to participate in this study.

## Author Contributions

The author confirms being the sole contributor of this work and has approved it for publication.

### Conflict of Interest

The author declares that the research was conducted in the absence of any commercial or financial relationships that could be construed as a potential conflict of interest.

## Footnotes

^1^Although multiple significance tests are reported, Bonferonni corrections were not deemed necessary as they are overly conservative and might lower Type 1 errors at the expense of increasing Type 2 errors. Instead, confidence intervals are provided, which can be anyways considered a superior alternative to significance testing (Brandstätter, 1999).
